# Sex-divergent effects on the NAD+-dependent deacetylase sirtuin signaling across the olfactory–entorhinal–amygdaloid axis in Alzheimer’s and Parkinson’s diseases

**DOI:** 10.1186/s13293-023-00487-x

**Published:** 2023-02-08

**Authors:** Paz Cartas-Cejudo, Mercedes Lachén-Montes, Isidro Ferrer, Joaquín Fernández-Irigoyen, Enrique Santamaría

**Affiliations:** 1 Clinical Neuroproteomics Unit, Proteomics Platform, Navarrabiomed, Hospitalario Universitario de Navarra (HUN), IdiSNA, Navarra Institute for Health Research, Universidad Pública de Navarra (UPNA), Irunlarrea 3, 31008 Pamplona, Spain; 2 Department of Pathology and Experimental Therapeutics, CIBERNED (Network Centre of Biomedical Research of Neurodegenerative Diseases), Bellvitge University Hospital/Bellvitge Biomedical Research Institute (IDIBELL), Institute of Health Carlos III, University of Barcelona, Hospitalet de Llobregat, Barcelona, Spain

**Keywords:** Sirtuin, Olfaction, Sexual dimorphism, Alzheimer, Parkinson, Proteomics

## Abstract

**Background:**

Smell impairment is one of the earliest features in Alzheimer’s (AD) and Parkinson’s diseases (PD). Due to sex differences exist in terms of smell and olfactory structures as well as in the prevalence and manifestation of both neurological syndromes, we have applied olfactory proteomics to favor the discovery of novel sex-biased physio-pathological mechanisms and potential therapeutic targets associated with olfactory dysfunction.

**Methods:**

SWATH-MS (sequential window acquisition of all theoretical fragment ion spectra mass spectrometry) and bioinformatic workflows were applied in 57 post-mortem olfactory tracts (OT) derived from controls with no known neurological history (*n* = 6F/11M), AD (*n* = 4F/13M) and PD (*n* = 7F/16M) subjects. Complementary molecular analyses by Western-blotting were performed in the olfactory bulb (OB), entorhinal cortex (EC) and amygdala areas.

**Results:**

327 and 151 OT differentially expressed proteins (DEPs) were observed in AD women and AD men, respectively (35 DEPs in common). With respect to PD, 198 DEPs were identified in PD women, whereas 95 DEPs were detected in PD men (20 DEPs in common). This proteome dyshomeostasis induced a disruption in OT protein interaction networks and widespread sex-dependent pathway perturbations in a disease-specific manner, among them Sirtuin (SIRT) signaling. SIRT1, SIRT2, SIRT3 and SIRT5 protein levels unveiled a tangled expression profile across the olfactory–entorhinal–amygdaloid axis, evidencing disease-, sex- and brain structure-dependent changes in olfactory protein acetylation.

**Conclusions:**

Alteration in the OT proteostasis was more severe in AD than in PD. Moreover, protein expression changes were more abundant in women than men independent of the neurological syndrome. Mechanistically, the tangled SIRT profile observed across the olfactory pathway-associated brain regions in AD and PD indicates differential NAD (+)-dependent deacetylase mechanisms between women and men. All these data shed new light on differential olfactory mechanisms across AD and PD, pointing out that the evaluation of the feasibility of emerging sirtuin-based therapies against neurodegenerative diseases should be considered with caution, including further sex dimension analyses in vivo and in clinical studies.

**Supplementary Information:**

The online version contains supplementary material available at 10.1186/s13293-023-00487-x.

## Highlights

- Quantitative proteomics is a useful approach to biochemically characterize the pathological neurodegeneration that occurs at the level of olfactory areas.
- Alteration in the olfactory tract proteostasis is more severe in AD than in PD.
- Protein expression changes are more abundant in women than men independent of the neurological syndrome.
- Functional enrichment analysis unveiled multiple common biological derangements between both sexes across both pathologies.
- Significant variations in the NAD+-dependent deacetylase sirtuin (SIRT) signaling, suggest sex, disease- and structure-specific changes in olfactory protein acetylation.

**Supplementary Information:**

The online version contains supplementary material available at 10.1186/s13293-023-00487-x.

## Background

The olfactory bulb (OB) is the first site for the processing of olfactory information in the brain. Axons from olfactory receptor neurons exit the olfactory epithelium (OE), grow toward the brain, and penetrate to the OB [1], where they synapse on the dendrites of mitral and tufted cells. The axons of these neurons then emerge from the OB, forming a discrete fiber bundle, the so-called olfactory tract (OT) [2]. These OT axons collateral branches to the olfactory cortex, where olfactory information is processed [3]. The piriform cortex is the largest and most distinctive olfactory cortical area; the periamygdaloid cortex, located ventrolateral to the pyriform cortex, and the rostral part of the entorhinal cortex also receive axons from the OB [3]. Interestingly, olfactory dysfunction is a common feature in Alzheimer’s (AD) and Parkinson’s diseases (PD) [4–6], being considered as a premature sign of neurodegeneration and consequently, a reliable marker [7]. An extensive analysis of the protein aggregates in OB and OT derived from post-mortem brains has revealed that the presence and severity of hyperphosphorylated Tau, Aβ, and α-synuclein pathology in both olfactory sites reflects the presence and severity of respective pathologies in other brain regions [8]. A significant degeneration of axons has been detected in the OT from AD subjects [9]. In pre-AD mild cognitive impairment (MCI) subjects, the loss of fiber OT integrity corresponds to a loss of gray matter density in parallel with a reduced glucose metabolism in central olfactory structures [10, 11]. Interestingly, OT undergoes early and sequential morphological alterations that correlate with the development of dementia [12]. On the other hand, atrophy, and changes in the structural integrity of OT has been also observed in PD subjects with respect to controls [13]. Besides, OT MRI diffusion measures are not adequate as early clinical biomarker for PD [14]. OT volume is smaller in PD than other movement disorders, such as Multiple system atrophy (MSA), Progressive supranuclear palsy (PSP), and Corticobasal degeneration (CBD) [15]. However, the comprehensive molecular profiling of the OT in human neurodegenerative disorders has received little attention. It is well-known that sex differences exist in terms of smell, olfactory structures, and olfactory-cell organization [16–19]. In addition, sex differences are also present in the prevalence and manifestation of neurological syndromes, such as AD [20–23] and PD [24–28]. The aim of this work was to apply mass spectrometry-based quantitative proteomics as a discovery platform to explore the magnitude of the OT proteome modulation in AD and PD as well as the sex-specific pathway modulation in this area. Multi-regional analysis across the olfactory route (encompassing the OB, OT, entorhinal cortex and amygdala structures) pointed out significant variations in the NAD+-dependent deacetylase sirtuin signaling, suggesting sex-, disease- and structure-specific changes in olfactory protein acetylation.

## Methods

### Materials

Antibodies for pERK (#4370), ERK (#9102), pAKT (#4060), AKT (#4685), pP38 (#9211), P38 (#9212), pNFkB (#3033), NFkB (#8242), pAMPK (#2535), AMPK (#2532), pFAK (#3281), FAK (#3285), SIRT1, SIRT2, SIRT3 and SIRT5 (#9787) and acetylated-Lysine (#9441S) Signaling were purchased from Cell Signaling. Antibody for PKCpan (SAB4502356) was purchased from Sigma Aldrich. Electrophoresis reagents were purchased from Biorad and trypsin from Promega. Antibodies and dilutions used in this study are shown in Additional file 1: Table S1.

### Human samples

Inform written consent from several Neurological Tissue Bank Services was obtained according to the Spanish Law 14/2007 of Biomedical Research for research purposes from relatives of patients included in this study. The study was conducted in accordance with the Declaration of Helsinki and all assessments, post-mortem evaluations, and procedures were previously approved by the Clinical Ethics Committee of Navarra Health Service (PID2019-110356RB-I00). Brain samples were obtained at post-mortem. One hemisphere was cut on coronal sections and small pieces of selected regions were rapidly dissected, immediately frozen, kept on labelled plastic bags, and stored at − 80 °C until use. The other hemisphere was fixed in 4% paraformaldehyde for about 4 weeks, and then cut on coronal sections. Samples of no less than 22 brain regions were embedded in paraffin. Sections 4 microns thick were obtained with a sliding microtome, de-waxed, and stained with haematoxylin and eosin, Klüver–Barrera, or processed for immunohistochemistry with the following antibodies: phospho-tau (AT8), β-amyloid, α-synuclein, TDP-43, p62, ubiquitin, GFAP, and Iba1. The neuropathological diagnosis was carried out according to the current neuropathological guidelines. Controls did not have suffered from neurological and mental diseases, and the neuropathological examination revealed no alterations excepting for small blood vessel disease; AD cases were categorized as middle and advanced Braak stages; and PD cases were identified as limbic or neocortical. Cases with associated proteinopathies (i.e., TDP-43 pathology) were not included in this study. The molecular studies were carried out using the available frozen samples of the contralateral hemisphere. Tissue samples of the OT, OB, amygdala, and entorhinal cortex were available from different individuals in the majority of cases. The lateral and medial part of the amygdala was chosen for study, but no sub-regions were obtained regarding the entorhinal cortex. Unfortunately, no tissue samples were available from the piriform cortex. Fifty-seven human OT samples from post-mortem subjects were employed for proteomics analysis, of which 17 of them were controls (*n* = 6F/11M; mean age ± SD 61.7 ± 10.5 years), 17 AD (*n* = 4F/13M; mean age ± SD 74.4 ± 7.8 years) and 23 PD subjects (*n* = 7F/16M; mean age ± SD 73.3 ± 7.7). Additional specimens from the OB, amygdala, and entorhinal cortex were considered for further molecular evaluations: OB (*n* = 8 per group, controls: 4W/4M, 71.2 ± 14.4 years; AD: 4W/4M, 78.3 ± 3.6 years; PD: 4W/4M, 79.6 ± 4.8 years); amygdala (*n* = 10 per group; controls: 3W/7M, 65 ± 10.1 years; AD: 4W/6M, 80.3 ± 2.5 years; PD: 2W/8M, 72.7 ± 9.9 years); entorhinal cortex (*n* = 10 per group; controls 5W/M, 69.1 ± 9.6 years; AD: 4W/6M, 78.1 ± 5.7 years; PD: 5W/6M, 79.3 ± 6.3 years). (Additional file 1: Table S1).

### OT preparation for proteomic analysis

Human OT samples were homogenized in lysis buffer containing 7 M urea, 2 M tiourea and 50 mM DTT. The homogenates were spinned down at 100,000×*g* for 1 h at 15 °C. Protein concentration was measured in the supernatants with the Bradford assay kit (BioRad). To increment the proteome coverage, in-solution and in-gel digestion workflows were carried out. In relation to in-solution digestion, pellet was dissolved in 6 M urea, 100 mM Tris, pH 7.8. Reduction was performed by adding DTT to a final concentration of 10 mM and incubation at 25 °C for 1 h. Subsequent alkylation was performed with 30 mM for 1 h in total dark. Then, an additional reduction step was executed with 30 mM DTT, allowing the reaction to stand at 25 °C for 1 h. The mixture was diluted to 0.6 M urea using MiliQ water. Hereafter, trypsin was added (Promega; 1:50, w/w) and the sample was incubated at 37 °C for 16 h. Digestion was quenched by acidification with acetic acid. The final step before mass-spectrometry was Vacuum Manifold platform. Then, samples dryness by vacuum centrifuge and resuspension in 10 µL of 2% acetonitrile, 0.1% formic acid and 98% miliQ water.

### SWATH–mass spectrometry proteomics: MS/MS library generation

A pool of 57 samples (1 µg/sample) derived from each OT human sample was used. Protein extracts were diluted in Laemmli sample buffer and loaded into a 4–15% stain free SDS–PAGE gel (BioRad). Total gel was stained with Coomassie Brilliant Blue and 13 equals slides from the pooled sample were excised from the gel and transferred into 1.5 mL Eppendorf tubes. Protein enzymatic cleavage was carried out with trypsin (Promega; 1:20, w/w) at 37 °C for 16 h. Peptide mixture was dried in a speed vacuum. Purification and concentration of peptides was performed with C18 Zip Tip Solid Phase Extraction (Millipore). Peptides recovered from in-gel digestion processing were reconstituted into a final concentration of 0.5 µg/µL of 2% ACN, 0.5% FA, 97.5% MilliQ-water prior to mass spectrometric analysis. MS/MS data sets for spectral library generation were acquired on a Triple TOF 5600+ mass spectrometer (Sciex, Canada) interfaced to an Eksigent nanoLC ultra 2D pump system (SCIEX, Canada) fitted with a 75 μm ID column (Thermo Scientific 0.075 × 250 mm, particle size 3 μm and pore size 100 Å). Before the separation, the peptides were concentrated on a C18 precolumn (Thermo Scientific 0.1 × 50 mm, particle size 5 μm and pore size 100 Å). Mobile phases were 100% water 0.1% formic acid (FA) (buffer A) and 100% Acetonitrile 0.1% FA (buffer B). Column gradient was developed in a gradient from 2% B to 40% B in 120 min. Column was equilibrated in 95% B for 10 min and 2% B for 10 min. During all process, precolumn was in line with column and flow maintained all along the gradient at 300 nL/min. Output of the separation column was directly coupled to nano-electrospray source. MS1 spectra was collected in the range of 350–1250 *m*/*z* for 250 ms. The 35 most intense precursors with charge states of 2 to 5 that exceeded 150 counts per second were selected for fragmentation, rolling collision energy was used for fragmentation and MS2 spectra were collected in the range of 230–1500 *m*/*z* for 100 ms. The precursor ions were dynamically excluded from reselection for 15 s. MS/MS data acquisition was performed using AnalystTF 1.7 (Sciex) and spectra files were processed through ProteinPilot v5.0 search engine (Sciex) using Paragon™ Algorithm (v.4.0.0.0) for database search. To avoid using the same spectral evidence in more than one protein, the identified proteins were grouped based on MS/MS spectra by the Progroup™ algorithm, regardless of the peptide sequence assigned. The protein within each group that could explain more spectral data with confidence was depicted as the primary protein of the group. FDR was performed using a non-lineal fitting method [29] and displayed results were those reporting a 1% Global FDR or better. The library generation-associated ProteinPilot group file was loaded into PeakView 2.1 and peaks from SWATH runs were extracted with a peptide confidence threshold of 99% threshold confidence (Unused Score ≥ 1.3) and FDR lower than 1%. ProteinPilot was also used to extract the MS/MS spectra of the assigned peptides, where only proteins with at least to unique peptides were considered.

### SWATH–mass spectrometry proteomics: quantitative analysis

Protein extracts (20 µg) from each sample were reduced by addition of DTT to a final concentration of 10 mM and incubation at room temperature for 30 min. Subsequent alkylation by 30 mM iodoacetamide was performed for 30 min in the dark. An additional reduction step was performed by 30 mM DTT, allowing the reaction to stand at room temperature for 30 min. The mixture was diluted to 0.6 M urea using MilliQ-water, and after trypsin addition (Promega) (enzyme:protein, 1:50 w/w), the sample was incubated at 37 °C for 16 h. Digestion was quenched by acidification with acetic acid. The digestion mixture was dried in a SpeedVac. Purification and concentration of peptides was performed using C18 Zip Tip Solid Phase Extraction (Millipore). The peptides recovered were reconstituted into a final concentration of 0.5 µg/µL of 2% ACN, 0.5% FA, 97.5% MilliQ-water prior to mass spectrometric analysis. For SWATH-MS-based experiments the instrument (Sciex TripleTOF 5600+) was configured as described by Gillet et al. [30]. Briefly, the mass spectrometer was operated in a looped product ion mode. In this mode, the instrument was specifically tuned to allow a quadrupole resolution of Da/mass selection. The stability of the mass selection was maintained by the operation of the Radio Frequency (RF) and Direct Current (DC) voltages on the isolation quadrupole in an independent manner. Using an isolation width of 16 Da (15 Da of optimal ion transmission efficiency and 1 Da for the window overlap), a set of 37 overlapping windows were constructed covering the mass range 450–1000 Da. In this way, 1 μL of each sample was loaded onto a trap column (Thermo Scientific 0.1 × 50 mm, particle size 5 μm and pore size 100 Å) and desalted with 0.1% TFA at 3 μL/min during 10 min. The peptides were loaded onto an analytical column (Thermo Scientific 0.075 × 250 mm, particle size 3 μm and pore size 100 Å) equilibrated in 2% acetonitrile 0.1% FA. Peptide elution was carried out with a linear gradient of 2 to 40% B in 120 min [mobile phases A: 100% water 0.1% formic acid (FA) and B: 100% Acetonitrile 0.1% FA] at a flow rate of 300 nL/min. Eluted peptides were infused in the mass spectrometer. The Triple-TOF was operated in swath mode, in which a 0.050 s TOF MS scan from 350 to 1250 *m*/*z* was performed, followed by 0.080 s product ion scans from 230 to 1800 *m*/*z* on the 37 defined windows (3.05 s/cycle). Collision energy was set to optimum energy for a 2+ ion at the center of each SWATH block with a 15 eV collision energy spread. The mass spectrometer was always operated in high sensitivity mode. The resulting ProteinPilot group file from library generation was loaded into PeakView® (v2.1, Sciex) and peaks from SWATH runs were extracted with a peptide confidence threshold of 99% confidence (Unused Score ≥ 1.3) and a FDR lower than 1%. For this, the MS/MS spectra of the assigned peptides was extracted by ProteinPilot, and only the proteins that fulfilled the following criteria were validated: (1) peptide mass tolerance lower than 10 ppm, (2) 99% of confidence level in peptide identification, and (3) complete b/y ions series found in the MS/MS spectrum. Only proteins quantified with at least two unique peptides were considered.

### Bioinformatics and statistical analysis

The quantitative data obtained by PeakView® were analyzed using Perseus software (version 1.6.15.0) [31] was used to perform statistical analysis and visualization of the obtained data. Unpaired Student’s *t* test was used for direct comparisons between controls, AD and PD. Statistical significance was set at *P* value lower than 0.05 in all cases and 1% peptide FDR threshold was considered. In addition, proteins were considered significantly differentially expressed when their absolute fold change was below 0.77 (down-regulated proteins) and above 1.3 (up-regulated proteins) in linear scale. Boxplots were performed with R software (v 4.1.2). The association of the differentially expressed proteins with specifically dysregulated regulatory/metabolic networks in OT human simples was analyzed using QIAGEN’s Ingenuity Pathway Analysis (IPA; QIAGEN Redwood City). This software calculates significance values (*P* values) between each biological or molecular event and the imported molecules based on the Fisher’s exact test (*P* ≤ 0.05). The IPA comparison analysis considers and reports hierarchically the signaling pathway rank according to the calculated *P* value. Metascape [32] was also used to extract biological information associated with proteome functionality using default settings (minimum overlap: 3; minimum enrichment: 1.5; *P* < 0.01).

### Western-blotting

Equal amounts of OT, OB, amygdala, and entorhinal cortex protein derived from human samples (5 µg) were resolved in 4–15% stain free SDS–PAGE gels (BioRad) and electrophoretically transferred onto nitrocellulose membranes using a Trans-blot Turbo transfer system (up to 25 V, 7 min) (BioRad). Membranes were probed with primary antibodies at 1:1000 dilution in 5% nonfat milk or BSA according to manufacturer instructions. After incubation with the appropriate horseradish peroxidase-conjugated secondary antibody (1:5000), the immunoreactivity was visualized by enhanced chemiluminescence (Perkin Elmer) and detected by a Chemidoc MP Imaging System (BioRad). Equal loading of the gels was assessed using stain-free imaging technology and by Ponceau staining. Therefore, protein normalization was performed by measuring total protein directly on the gels used for western blotting, as previously described [33]. After densitometric analyses (Image Lab Software Version 5.2; Bio-Rad), optical density values were expressed as arbitrary units and normalized to total protein levels in each gel lane.

## Results

### Sex-dependent OT proteome alterations in AD and PD

**Fig. 1:**
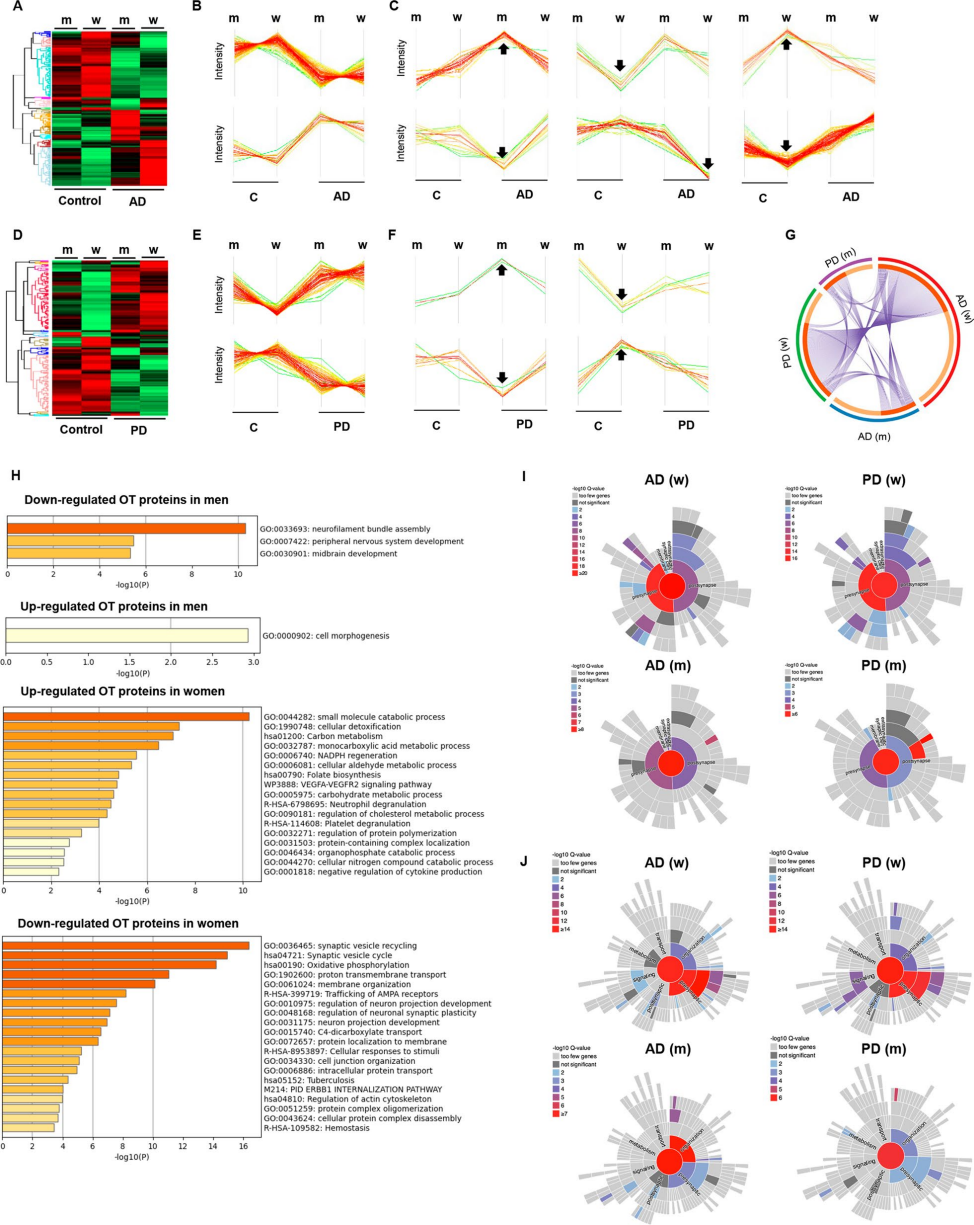
Sex differences in the OT proteome in AD and PD. **A** Heatmap representing the differential OT proteotyping in AD across sexes. **B** Sex-independent protein clusters specifically modulated in AD. **C** Sex-dependent protein clusters modulated in controls or in AD subjects. **D** Heatmap representing the differential OT proteotyping in PD across sexes. **E** Sex-independent protein clusters specifically modulated in PD. **F** Sex-dependent protein clusters modulated in controls or in PD subjects. **G** Circos-plot representing the OT deregulated proteome shared between AD and PD across sexes. On the inside, dark orange color represents the proteins that appear in multiple data sets and light orange color represents unique deregulated proteins specific of each experimental group. Purple lines indicate the proteome that is shared across biological conditions. **H** Functional clustering of proteins commonly deregulated in AD/PD men or AD/PD women. **I** Synaptic ontology analysis (subcellular distribution) of OT deregulated proteomes. **J** Synaptic ontology analysis (molecular function) of OT deregulated proteomes (m: men; w: women)

To characterize the OT site-specific proteomic signature in AD and PD across both sexes, a SWATH-MS-based approach was performed on OT specimens derived from controls with no known neurological history, AD and PD subjects (*n* = 57) (Additional file 1: Table S1 and Additional file 7: Fig. S1). Among 1835 quantified proteins across all experimental groups, we initially observed that protein subsets were not only differentially modulated by pathological phenotypes (Fig. 1A, B, D, E) but also in a sex-dependent manner (Fig. 1C–F). Interestingly, specific proteins tend to be modulated between men and women in the control group (Fig. 1C–F), indicating the importance of considering the sex dimension to map sex-specific differences in olfactory signaling. Moreover, we observed that part of the differential OT proteome was commonly altered between AD and PD (Fig. 1G), revealing shared molecular events that contribute to common mechanisms during olfactory neurodegeneration. Specifically, proteins involved in neurofilament bundle assembly (GO:0033693) were commonly down-regulated in men independent of the neurological phenotype (Fig. 1H). An overexpression of protein mediators in cell morphogenesis (GO:0000902) was particularly observed in men (Fig. 1H). However, an up-regulation of proteins related to small molecule catabolism and detoxification (GO:0044282; GO:1990748) and a drop in proteins related to synaptic vesicle recycling (GO:003645) were commonly observed in OTs derived from women, independent of the presence of AD or PD pathologies (Fig. 1H). A more detailed analysis of synaptic ontologies confirmed a severe presynaptic and postsynaptic disruption in women than in men (Fig. 1I); however, no significant differences at the level of synaptic components or synaptic biological process were found between AD and PD women (Fig. 1I, J).

### Functional commonalities and differences associated with sexual dimorphism in AD and PD

**Fig. 2:**
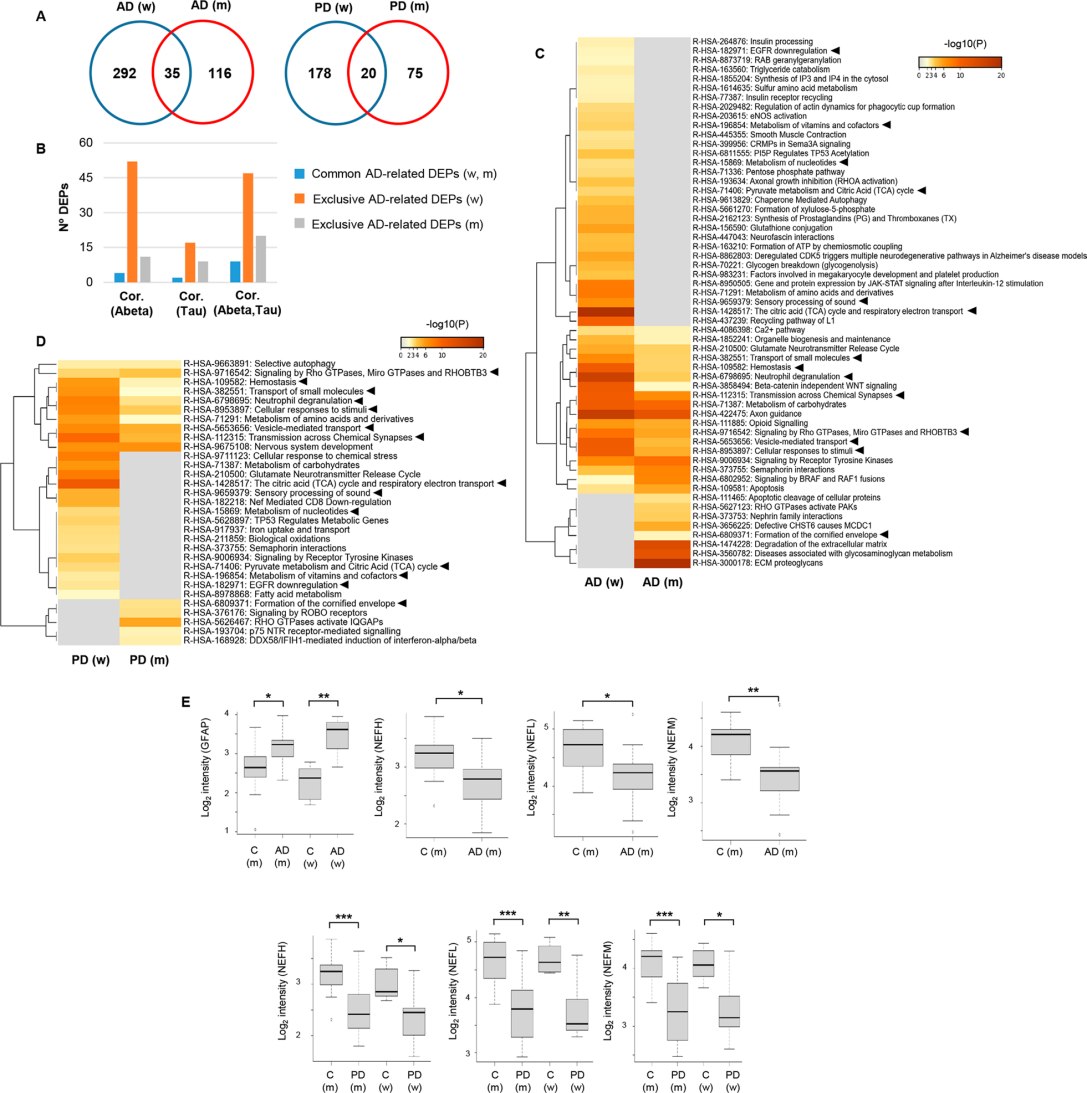
Functional impact of the deregulated OT proteostasis in AD and PD across both sexes. **A** Number of common and dissimilar deregulated proteins between men and women in each neurological disorder. **B** Integrative analysis of our OT proteome data derived from AD subjects with the brain region-specific omics data sets from AlzData database (http://www.alzdata.org/) [34]. **C** Functional mapping of disrupted OT proteome in AD women and men. **D** Functional mapping of disrupted OT proteome in PD women and men. Black triangles indicate biological processes commonly altered in AD and PD between both sexes. **E** Mass spectrometry-based quantitation of astrogliosis and axonal damage markers (m: men; w: women)

Our data revealed that the OT proteostatic imbalance was more severe in AD than in PD. 327 and 151 OT differentially expressed proteins (DEPs) were observed in AD women and AD men, respectively (35 DEPs in common) (Fig. 2A). In contrast, 198 and 95 DEPs were identified in PD women and PD men, respectively (20 DEPs in common) (Fig. 2A and Additional file 2: Table S2 and Additional file 3: Table S3). According to AlzData (http://www.alzdata.org/) [34], part of the differential OT proteome observed in our AD cohort showed a gene expression correlation with AD pathology in Aβ and/or Tau line AD mouse models (Fig. 2B). In addition, common deregulated proteins in AD women and men (TAGLN3, PDE2A, GAP43, CAMK2A, HPCAL4, CAMK2B, SGIP1, CAMK2G, YWHAG, DPYSL4, VIM, LGALS3, HSPB1 and GFAP) are differentially expressed in AD mouse models before AD pathology appears (Additional file 4: Table S4), which may be considered early alterations during the neurodegenerative process.

Although the number of commonly deregulated proteins in AD and PD between women and men was relatively low (Fig. 2A), functional enrichment analysis unveiled multiple common biological derangements between both sexes across both pathologies (Additional file 5: Table S5 and Additional file 6: Table S6). In relation to AD, OT significant alterations related to organelle biogenesis, glutamate release cycle, metabolism of carbohydrates, axon guidance and semaphorin signaling between others were commonly mapped in women and men (Fig. 2C). However, multiple disrupted significant pathways corresponded to sex-dependent specificities, such as recycling of adhesion molecule L1 and Jak-STAT signaling in AD women, and proteoglycan metabolism and extracellular matrix degradation in AD men (Fig. 2C). With respect to PD, pathways involved in selective autophagy and aminoacid metabolism were imbalanced in women and men (Fig. 2D). In addition, part of the enriched pathways exclusively modulated in PD women were chemical stress response, metabolism of carbohydrates and glutamate cycle, while signaling by ROBO receptors and IQGAPs activation by RHO GTPases were processes specifically disrupted in PD men (Fig. 2D). As indicated in Fig. 2C, D, functional commonalities were observed not only in AD and PD, but also across sexes at the level of the OT.

### OT sex-dependent effects in protein complexes, functional interactomes and signaling dynamics in AD and PD

**Fig. 3:**
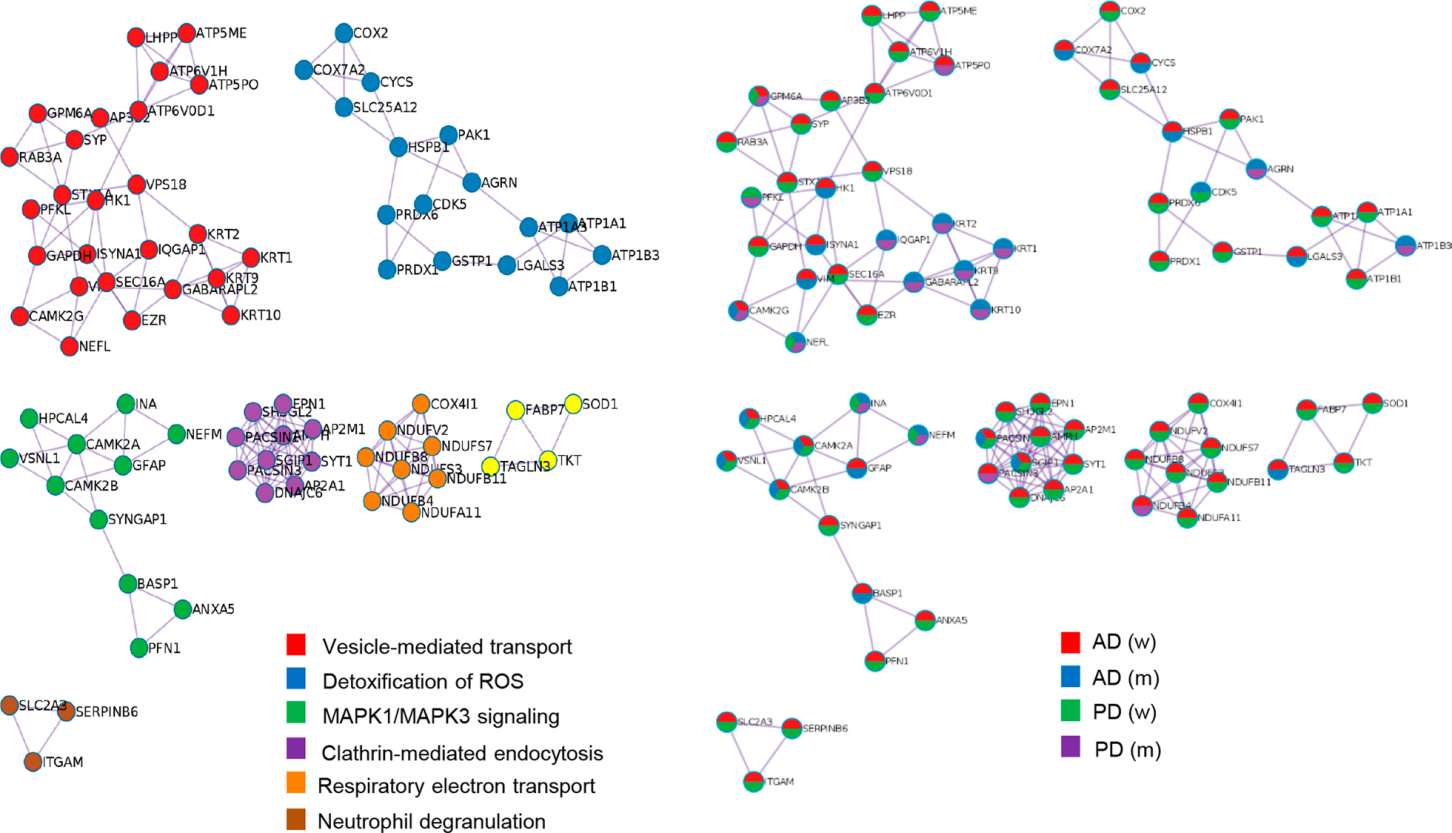
Sex influences on the composition of the OT protein complexes differentially modulated in AD and PD. Protein complexes embedded in OT proteomics outputs were automatically extracted by the MCODE algorithm [35]. Through Metascape tool [32], three most significantly enriched ontology terms were combined to annotate putative biological roles for each MCODE complex (left). Protein components of each complex differentially modulated in AD and PD considered in our survey (lower) (m: men; w: women)

**Fig. 4:**
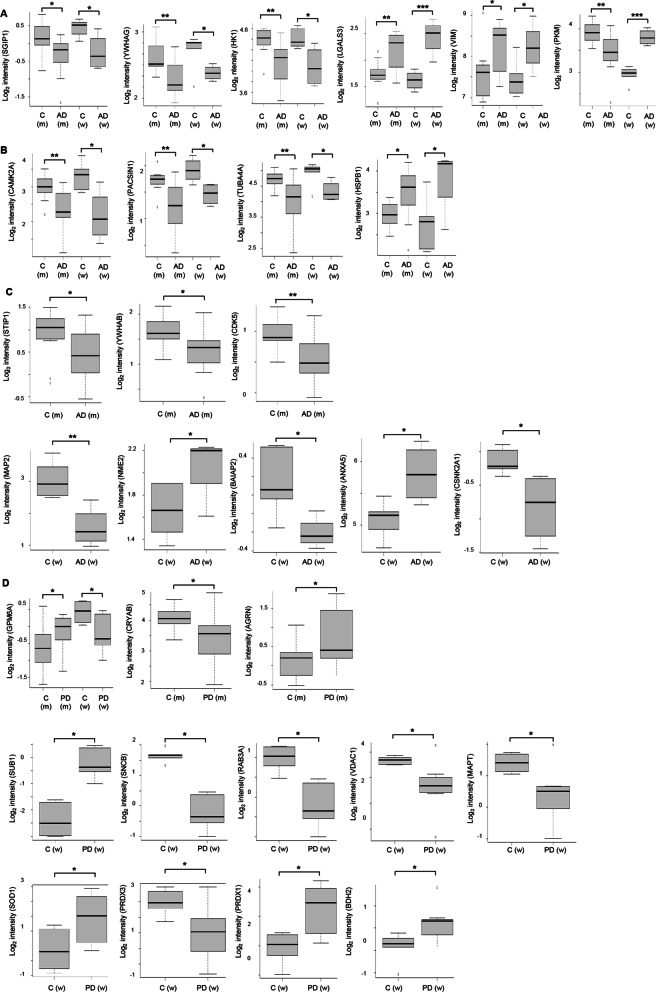
Sex-dependent changes in the constitutive interactome of neuropathological substrates in AD and PD. Experimentally demonstrated protein interactors of human APP, Tau and α-synuclein were obtained from Biogrid database [36]. Mass spectrometry-based protein intensity of deregulated APP interactors (**A**), Tau interactors (**B**), shared APP and Tau interactors (**C**) and α-synuclein interactors (**D**)

**Fig. 5:**
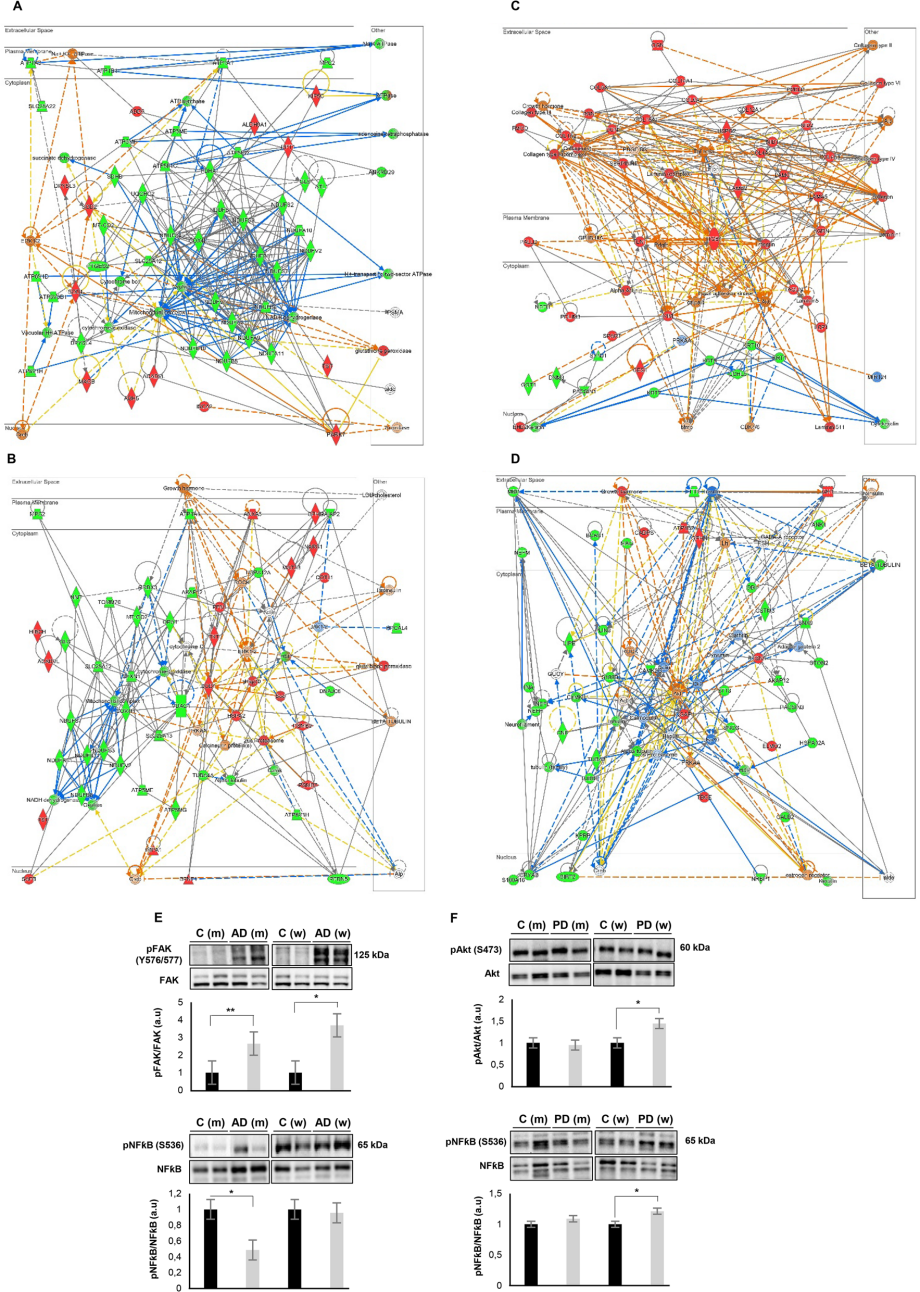
Differential impact of sex in OT protein networks and signaling dynamics in AD and PD. **A** Functional networks associated with AD women (**A**), PD women (**B**), AD men (**C**) and PD men (**D**). AKT, FAK and NFkB activation state in the OT derived from AD (**E**) and PD (**F**) subjects

Differentially expressed markers of astrogliosis and axonal damage were detected by olfactory proteomics. GFAP was significantly up-regulated in AD (Fig. 2E), although a non-significant tendency to up-regulation was also observed in both PD groups (PD M vs. C M: FC: 1.28; *P*-val: 0.14; PD F vs. C F: FC 1.57; *P*-val: 0.07) (Additional file 3: Table S3). The heteropolymeric components of the neurofilaments (NEFH, NEFL, NEFM) were specifically down-regulated in AD men (Fig. 2E). However, protein levels of the neurofilament subunits were significantly decreased in both sexes from the PD cohort (Fig. 2E). From the point of view of protein complexes, specific molecular clusters derived from the OT proteomic data sets were differentially targeted by the neurodegenerative process associated with AD and PD. According to the MCODE algorithm [35], part of the protein machinery involved in vesicle-mediated transport and clathrin-mediated endocytosis was differentially targeted by both sexes independent of the neurodegenerative disease (Fig. 3). We wanted to know the impact of the OT neurodegeneration at the level of APP, Tau and α-synuclein interactors. For that, only experimentally demonstrated information present in Biogrid repository was considered [36]. Protein levels corresponding to APP interactors such as SGIP1, WYHAG, HK1, LGALS3 and VIM were commonly deregulated in AD women and men. However, PKM levels were inversely regulated between AD men and women (Fig. 4A). With respect to Tau interactors, CAMK2A, PACSIN1, TUB4A were significantly reduced in AD across both sexes, whereas OT HSPB1 levels were increased in both sexes (Fig. 4B). Sex-specificities were observed for shared interactors between APP and Tau. STIP1, WYHAB and CDK5 were exclusively altered in AD men and protein levels corresponding to MAP2, NME2, BAIAP2, ANX5 and CSNK2A1 were differentially expressed in AD women (Fig. 4C). With respect to α-synuclein interactors, only GPM6A was commonly altered in PD across both sexes (Fig. 4D). CRYAB and AGRN were only altered in PD men, whereas SUB1, SNCB, RAB3A, VDAC1, MAPT, SOD1, PRDX3, PRDX1 and BDH2 protein levels were exclusively modified in PD women (Fig. 4D). Furthermore, network-driven OT proteomics also revealed sex-dependent effects at subcellular level independent of the neurological background. High-scoring functional interactome associated with AD (Fig. 5A) and PD women (Fig. 5B) impacted on mitochondrial respiratory chain complexes. On the other hand, high-scoring deregulated protein networks in AD and PD men were directly involved in extracellular and intracellular cytoskeleton imbalance, respectively (Fig. 5C, D). All these analyses suggest that part of the differential mechanisms between women and men during the loss of the OT molecular integrity are organelle-specific and may be triggered by fluctuations on the functional interactomes corresponding to classical neuropathological substrates (Additional file 8: Fig. S2 and Additional file 9: Fig. S3).

**Fig. 6:**
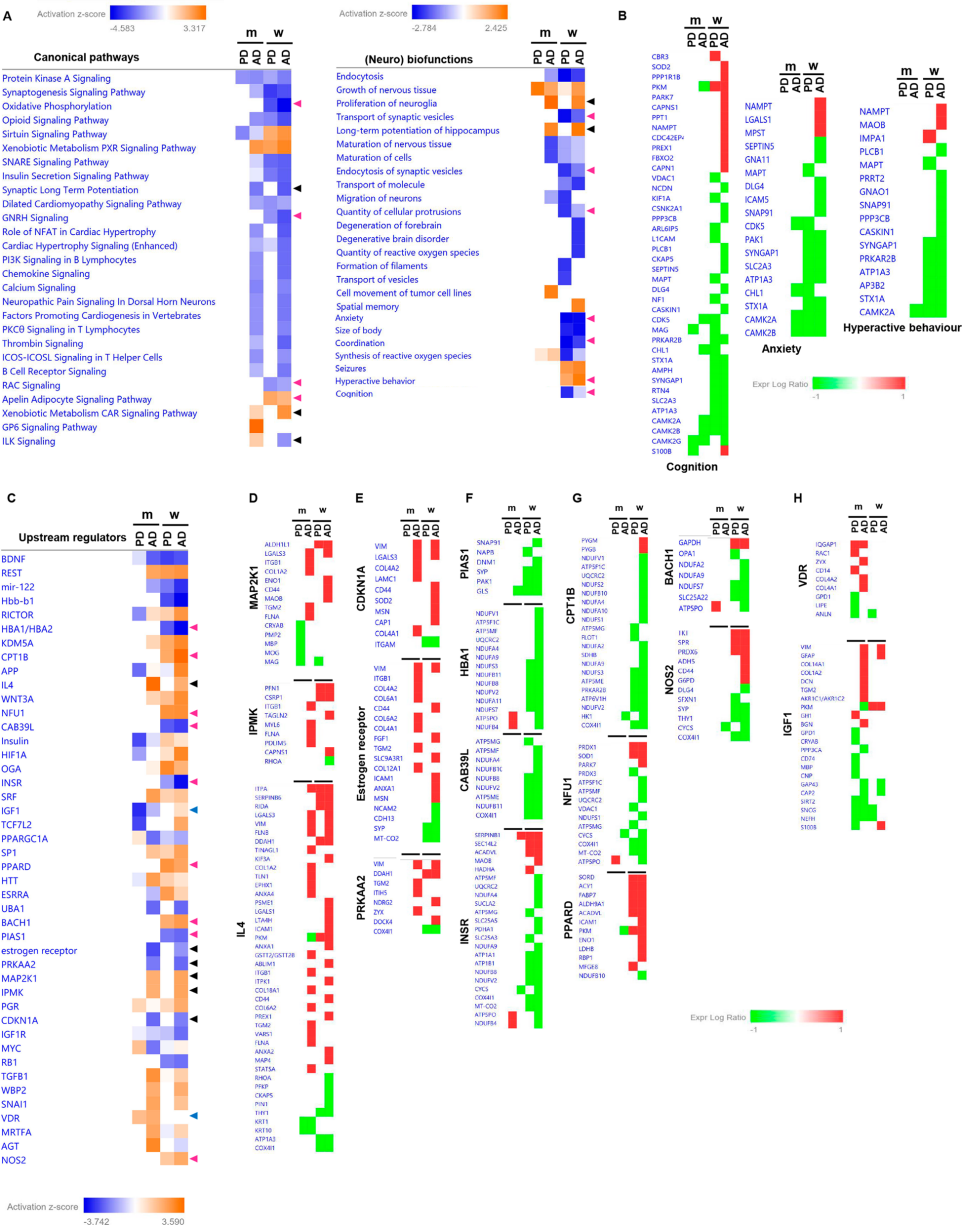
Predictive activation profile of pathways, biofunctions and upstream regulators at the level of OT in AD and PD. Based on OT proteomic data sets, Systems Biology analysis were performed through the Ingenuity Pathway Analysis software [100]. Activation prediction of significantly altered pathways and neuronal functions (**A**, **B**) as well as upstream regulators (**C**–**H**). The activation *z*-score is calculated as previously described [100]. It makes predictions about potential regulators using information about the direction of protein regulation and comparing with a model that assigns random regulation directions. Blue and orange squares indicate inhibition and activation directionality, respectively. Black triangles refer to processes/molecules with an activation score exclusively associated with AD. Pink and blue triangles indicate processes/molecules with an activation profile specifically associated with women or men, respectively. Red: up-regulation; green: down-regulation; m: men; w: women)

Previous reports point out that the activation dynamics of survival routes is compromised in AD and PD at the level of the OB [37, 38]. Interestingly, we observed a divergence in the activation profile of specific kinases and transcription factors depending on pathology and/or sex. FAK activation was specifically increased in AD OTs, while an over-activation of Akt was exclusively observed in PD women (Fig. 5E, F). However, OT NFkB activity was down-regulated in AD men and up-regulated in PD women (Fig. 5E, F). No changes were observed for ERK1/2, p38 MAPK and AMPK across sexes in both pathologies at OT level (Additional file 10: Fig. S4). A System biology approach also pointed out potential sexually dimorphism changes in the activation state of additional signaling pathways and upstream regulators across AD and PD phenotypes (Fig. 6). Based on differential protein expression profiles, oxidative phosphorylation, GNRH signaling and RAC signaling present an inhibitory trend specifically in AD and PD women (Fig. 6A). In addition, OT proteotypes indicated that neuronal biofunctions such as transport and endocytosis of synaptic vesicles, anxiety, coordination and cognition tend to be inhibited in women independent of the neuropathological disorder, whereas hyperactive behavior showed an activation profile (Fig. 6A, B). Upstream regulator-dependent analysis also revealed that differential OT proteomes pointed out signaling molecules activated or inhibited in AD (Fig. 6C–E), specifically inhibited or activated in women (Fig. 6C, F, G) or specifically modulated in men (Fig. 6C, H). Collectively, these data suggested that sex influences not only at olfactory proteostatic level but also in the crosstalk signaling.

### Sirtuin signaling is differentially altered across the olfactory bulb–olfactory tract–amygdaloid–entorhinal axis in a sex-dependent manner: divergency between AD and PD

**Fig. 7:**
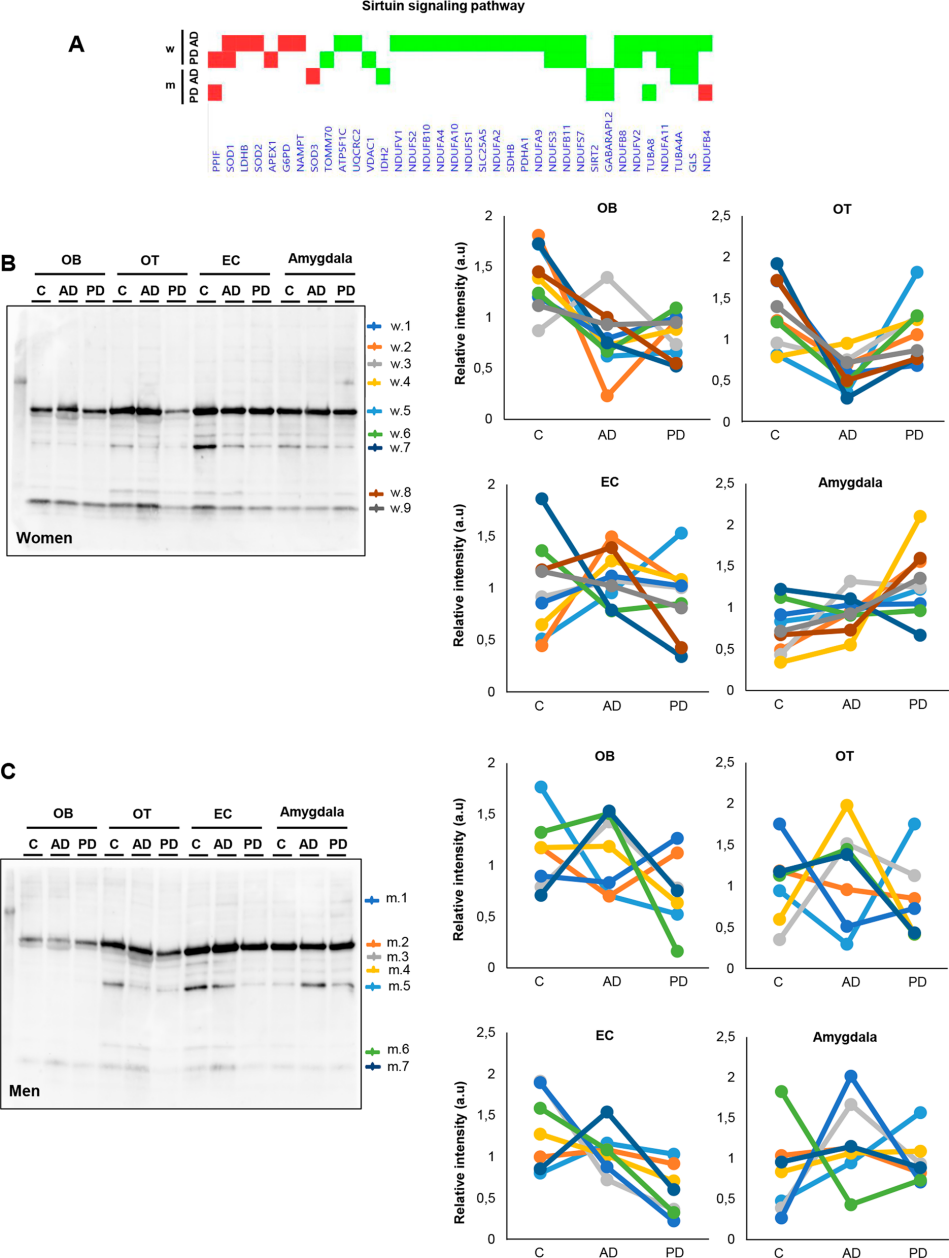
Differential acetylome across the olfactory axis in AD and PD. **A** Deregulated OT protein expression profile related to SIRT signaling pathway (red: up-regulation; green: down-regulation; m: men; w: women). **B** Western-blotting against Lys-acetylated proteins at the level of OB, OT, EC and amygdala in AD and PD women. Sample pooling (*n* = 5/group) was used in all biological conditions. Equal loading of the gels was assessed by stain-free digitalization. Right panels indicate relative intensity levels for multiple bands (w.1 to w.9 indicated on the blot) normalized by total protein in each gel lane across OB, OT, EC and amygdala protein extracts. **C** Western-blotting against Lys-acetylated proteins at the level of OB, OT, EC and amygdala in AD and PD men. Sample pooling (*n* = 5/group) was used in all biological conditions. Equal loading of the gels was assessed by stain-free digitalization. Right panels indicate relative intensity levels for multiple bands (m.1 to m.7 indicated on the blot) normalized by total protein in each gel lane across OB, OT, EC and amygdala protein extracts

**Fig. 8:**
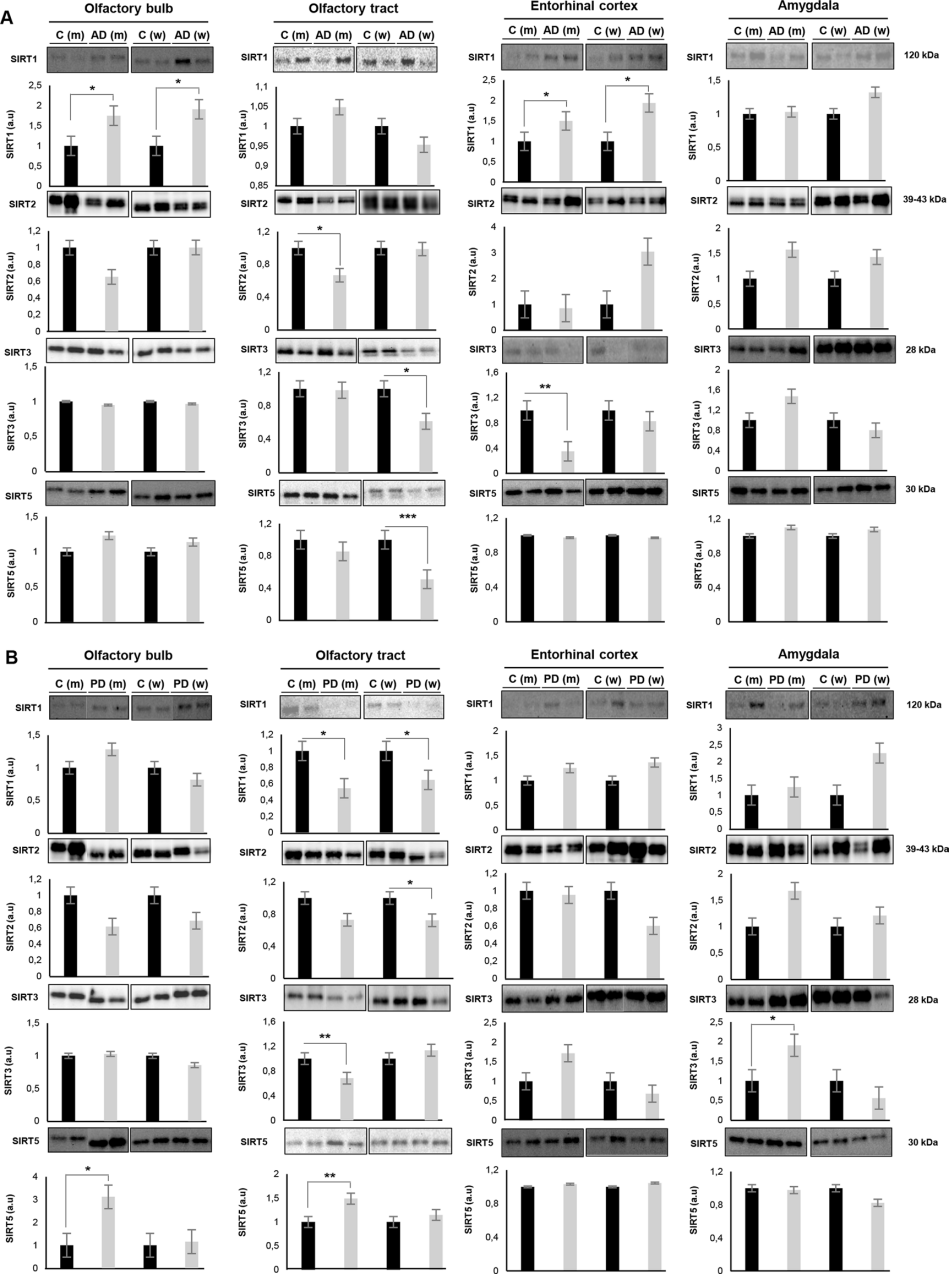
Differential sex-associated changes in Sirtuin (SIRT) signaling across the olfactory bulb, olfactory tract, entorhinal cortex and amygdala in AD and PD. **A** Protein expression of SIRT family (SIRT1, 2, 3 and 5) across the olfactory axis in AD. **B** Protein expression of SIRT family (SIRT1, 2, 3 and 5) across the olfactory axis in PD. Western-blotting were performed in *n* = 2–6/group/structure (Additional file 1: Table S1). Representative images are shown. Equal loading of the gels was assessed by stain-free digitalization. Panels show histograms of band densities. Data are presented as mean ± SEM. *P < 0.05 vs. control group; **P < 0.01 vs. control group (a.u: arbitrary units)

One of the signaling routes that presented an olfactory sex-dependent performance across AD and PD was the sirtuin (SIRT) pathway (Figs. 6A and 7A). Moreover, experimentally demonstrated SIRT interactors were also differentially expressed at the level of the OT across both disorders (Additional file 11: Fig. S5). Owing to the intrinsic role of this route in switching between deacetylation and acetylation processes [39], acetylome variations were checked across AD and PD groups by Western-blotting using an anti-acetylated lysine antibody in a multi-regional format, covering the OB, OT, entorhinal cortex (EC) and amygdala areas. The high-abundant acetylome varied across olfactory-related areas in AD and PD (women and men) (Fig. 7B, C), detecting global significant changes across the OB-OT area (Additional file 12: Fig. S6). To increase our understanding of the specific role of SIRTs in olfactory neurodegeneration, the SIRT protein expression profile was independently monitored across AD and PD women and men through the olfactory axis. As shown in Fig. 8, SIRT1 was up-regulated at the level of the OB and EC in AD men and women (Fig. 8A). In contrast, SIRT1 expression was exclusively decreased at the level of the OT in PD women and men (Fig. 8B). SIRT2 protein levels were dropped in OT from AD men and in PD women (Fig. 8A, B). With respect to SIRT3, its protein expression was significantly reduced in the OT from AD women and PD men. A specific SIRT3 increment was also observed in the amygdala from PD men (Fig. 8B). SIRT5 protein levels were significantly down-modulated in the OT from AD women, whereas a significant increase was evidenced in the OB-OT areas in PD men (Fig. 8B). All these data pointed out that the dyshomeostatic SIRT signaling that accompany the neurodegenerative process differently impacts depending on sex, showing disease-specificities and brain structure-dependencies, being the OT the most affected area in both syndromes.

## Discussion

The consideration of sex dimension is pivotal to improve our understanding of neurodegeneration and the biological mechanisms that contribute to the etiology, manifestation, and potential therapeutics of neurological syndromes [40]. In fact, multiple health incongruities in therapeutic and diagnostic fields have been associated with the lack of inclusion of women in basic research as well as women in clinical trials [41, 42]. Due to smell deficit has been proposed as an early indicator of AD and PD [43] and sex differences exist in terms of olfactory functionality [16–19], we consider that a better comprehension of the molecular mechanisms disrupted at olfactory level in a sex-dependent manner might offer unknown targets for earlier diagnosis and therapeutic intervention in neurological disorders. Up to now, the OT characterization has been mainly addressed in two different contexts. In humans, electrophysiological testing, together with morphological imaging approaches are used to evaluate the OT in the clinical assessment of the sense of smell [44]. In mice, the OT is broadly studied as a model to understand the mechanisms underlying the guidance of growing axons [7]. In this work, we report that: (i) OT proteomic alteration is more severe in AD than in PD; (ii) this OT proteostatic imbalance is higher in women than in men independent of the disease; (iii) despite the dissimilar molecular profiles unveiled between women and men, common protein intermediates and biological pathways have been identified across both diseases; (iv) experimentally demonstrated protein interactors of canonical neuropathological substrates are differentially modulated across both sexes at OT level; (v) specific olfactory signaling routes governed by NFkB are differentially modulated across sexes in AD and PD; (vi) sex-dependent protein acetylation changes are evidenced across the olfactory axis in both diseases and (vii) sex-specific sirtuin signaling imbalance occurs across the olfactory axis in AD and PD, in which protein expression changes of sirtuin family members at the level of OT are extensively more dynamic. All these sex-specific molecular profiles provide novel mechanistic clues on the divergent mechanisms involved in the olfactory neurodegeneration that occur in AD and PD [6].

The minimal overlap observed in OT proteome remodeling between women and men (in both diseases) support the hypothesis that distinctive pathophysiological processes are involved in the olfactory neurodegenerative process. This argument is clearly supported when the neuropathological stage is also considered (Additional file 13: Fig. S7). Although, common pathways are disrupted in AD advanced stages (Braak V–VI) and PD advanced stage (neocortical stage) independent of the sex variable, multiple biofunctions are specifically altered in a sex- and neuropathological stage-dependent manner (Additional file 13: Fig. S7). Due to the inherent limitations of our sample cohort, further research is needed to analyze the dual effect of sex and neuropathological grading in initial phases of AD and PD at olfactory level. SIRTs correspond to class III histone deacetylase enzymes (found throughout different cellular compartments) targeting both histone and non-histone substrates involved in metabolism, myelination and apoptosis [45–47]. From a neuropathological point of view, although SIRTs have been previously associated with multiple neurological syndromes [39, 48, 49], sex dimension has not been broadly considered. We have observed a tangled sex-dependent SIRT protein expression profile (SIRT1, 2, 3 and 5) that significantly differ in AD and PD across primary and secondary olfactory areas. Specifically, SIRT1 was increased in the OB and EC in AD women and men. It has been shown that SIRT1 protects neuronal axons, induces neurite outgrowth and regulates long-term potentiation and neurogenesis [50, 51]. SIRT1 levels are brain region-dependent in AD [49], being able to degrade the Aβ peptide in primary astrocytes and to reduce ROS and peroxidation levels in APP/PS1 AD model, decreasing senile plaques and improving learning and memory activities [52–54]. In addition, SIRT1 also interferes with Tau metabolism [55], reducing the acetylated tau levels and avoiding the propagation of pathological Tau [56, 57]. All these beneficial effects, together with the up-regulation of multiple neurotrophic factors (BDNF, GDNF and VEGF) by SIRT1 [58], could indicate a general neuroprotective mechanism through the olfactory axis in AD, specially at the level of the OB and EC. However, we observed a specific drop in SIRT1 levels in the OT derived from PD subjects. It has been demonstrated that SIRT1 activity slows down when extracellular α-synuclein is present as well as in PD–post-mortem brain material [59, 60]. Moreover, pharmacological activation of SIRT1 triggers α-synuclein degradation through the deacetylation of LC3 and up-regulation of LC3-II [61]. Interestingly, it is known that SIRT1 presents known roles in recovery olfactory function. Specifically, SIRT1 is involved in olfactory function maintenance through the protection of subventricular zone-derived neural stem cells from DNA Double-Strand Breaks [62]. Moreover, the increment in olfactory bulb SIRT1 expression is associated with the recovery of olfactory function under bulbar excitotoxic insults [63]. Despite multiple evidence point out that SIRT1 is an olfactory-promoting factor and neuroprotective in different neurodegenerative contexts, it seems obvious that its positive role may depend on multiple factors such as oxidative stress levels [64] and the neuropathological damage across the olfactory axis.

Although SIRT2 temporal cortical levels are increased in AD patients [65], we observed a specific significant decrease in the OT from AD men. In general, and in direct opposition to SIRT1, reduced expression levels of SIRT2 tend to be beneficial in the context of AD. In vitro and in vivo studies have revealed that low SIRT2 levels diminish Aβ toxicity [65–67] and Tau phosphorylation [68]. This conclusion has been also corroborated using SIRT2 inhibitors in several AD models [69, 70]. With respect to PD, despite SIRT2 levels are unchanged in the substantia nigra pars compacta of PD subjects [71], OT SIRT2 levels were down-regulated in PD women. Experimental data of SIRT2 expression in PD models are significantly divergent [72, 73]. Due to acetylation induces a reduction in α-synuclein oligomerization and aggregation, and SIRT2 interacts with alpha-synuclein leading to its deacetylation [74, 75], SIRT2 inhibition has been proposed as a potential therapeutics against synucleinopathies [76, 77].

SIRT3 expression diminishes in cortical regions from APP/PS1 AD model as well as in AD patients [78–80]. In our case, a significant decrease was observed in OT from AD women and EC from AD men. Although no correlation has been observed between SIRT3 and Aβ plaques across different human brain areas [49], SIRT3 activation protects neurons from Aβ toxicity [81–83] and reduces Tau and acetylated Tau [84], probably through a mitochondrial-related energetic route [85, 86]. In relation to PD, a decrease in SIRT3 levels was observed in the OT from PD men, whereas SIRT3 increment was evidenced in the amygdala from the same group. SIRT3 reduction increases ROS production and α-synuclein aggregation, triggering the loss of dopaminergic neurons [87, 88]. Moreover, SIRT3 overexpression impacts on mitochondrial bioenergetics, mitigating the oxidative stress through the SIRT3-mediated deacetylation of Mn-SOD [89, 90] and increasing the autophagic capacity through the LKB1–AMPK–mTOR pathway, inducing ROS alleviation and inhibiting alpha-synuclein accumulation [91]. We have evidenced a significant drop in SIRT5 levels in the OT from AD women as well as an increment in protein levels at the level of OB and OT from AD and PD men. Unlike other SIRTs, SIRT5 is involved in deacetylation, demalonylation, deglutarylation and desuccinylation processes [64]. Specifically, the IDH2 desuccinylation and the G6PD deglutarylation by SIRT5 increase cellular antioxidant mechanisms [92]. It has been shown that a SIRT5 deficiency triggers ROS overproduction and mitochondrial imbalance inducing a motor dysfunction in a PD model [93], suggesting a neuroprotective effect.

Although dysregulation of histone acetyltransferases (HATs) and deacetylases (HDACs) has been considered fundamental for the onset and/or progression of neurological disorders [94], the differential spatial dysregulation observed in the HDAC SIRT protein family suggests that protein deacetylation-based mechanisms involved in neuronal homeostasis and proteostasis of proteotoxic species differs not only between AD and PD but also between women and men. Additional research is needed to elucidate the sexually dimorphic specificity of SIRT protein targets in aggregate-forming neurological disorders to promote the development of novel compounds with therapeutic potential that tightly modulate protein acetylation.

## Limitations of the study

Although many intricacies in OT molecular homeostasis have been uncovered, there are potential limitations that warrant discussion. We cannot discard that part of the molecular alterations identified in this study may be age-dependent and not strictly associated with the neurodegenerative process. Moreover, due to technological issues, we failed to accurately quantify part of the low-abundant proteome as well as hydrophobic proteins and receptors that might also be involved in the olfactory neurodegeneration. Moreover, our OT data are limited by protein abundance averaging among multiple cell types present at the level of the OT, complicating the characterization of olfactory cell-type-specific molecular alterations. In a similar way, SIRT protein levels were monitored in bulk tissue, hampering the SIRT distribution evaluation and correlation across cell types. Finally, we cannot rule out the possibility that other SIRT members not considered in our study may be part of sex-dependent deacetylation regulatory mechanisms involved in olfactory neurodegeneration. Further acetylome studies are needed to decipher the substrate specificity associated with each SIRT across the olfactory axis as well as the underexplored sex-dependent acetylation changes that occur in target brain structures associated with the neurodegenerative process.

## Perspective and significance

In general, the role of each SIRT protein form has been analyzed from a disease-centric perspective, leaving out the sexual factor. A hypothetical model has been proposed in which advantageous effects promoted by SIRTs could be presented in a sex-nonspecific manner. However, after reaching reproductive maturity, it has been hypothesized that SIRT dependency in modulating metabolic networks are evolutionarily decoupled from female longevity [95]. In this work, the neurodegenerative process differentially impacts on SIRT profile depending on disease, sex, and olfactory-related areas, affecting the global acetylation/deacetylation machinery of the human brain. Based on our sex-dependent data, and because of the complex pattern of temporal and spatial SIRTs expression across the brain [96], the lack of knowledge regarding the substrate specificity of each SIRT isoform in healthy and diseased conditions [97] and the recent growing interest in the deployment of SIRT modulators to ameliorate cognitive deficits and treat neurodegenerative diseases [39, 98, 99], additional research is needed to address the importance of sex differences in animal models and human trials in the evaluation of emerging sirtuin-based therapies against neurological syndromes.

## Supplementary Information

**Additional file 1: Table S1.** Antibody dilutions and human samples used in this study (PMI: post-mortem interval; W: women, M; men).

**Additional file 2: Table S2.** Differential OT proteins detected in AD.

**Additional file 3: Table S3.** Differential OT proteins detected in PD.

**Additional file 4: Table S4.** Interlocking of OT AD proteomics data sets and ALZData repository.

**Additional file 5: Table S5.** Functional analysis of OT DEPs in AD using Reactome database through Metascape tool.

**Additional file 6: Table S6.** Functional analysis of OT DEPs in PD using Reactome database through Metascape tool.

**Additional file 7: Figure S1.** Sex-dependent clustering based on the olfactory tract (OT) protein expression profiles derived from AD (A) and PD (B) subjects. Heatmap representation showing both clustering and the intensity for the OT proteins in each biological condition (ANOVA *p* value).

**Additional file 8: Figure S2.** Deregulated protein interactome associated with MAPT (Tau) in AD generated by IPA software. Green and red indicate down and up-regulated proteins, respectively. Orange and blue indications are activation or inhibitory mechanisms proposed by the IPA algorithm.

**Additional file 9: Figure S3.** Deregulated protein interactome associated with SNCA (α-synuclein) in PD generated by IPA software. Green and red indicate down and up-regulated proteins, respectively. Orange and blue indications are activation or inhibitory mechanisms proposed by the IPA algorithm.

**Additional file 10: Figure S4.** Western-blotting analysis of several survival kinases that are not modified in AD (A) or PD (B) at the level of the OT. Representative blots are shown.

**Additional file 11: Figure S5.** Exploration of human SIRT interactomes. Overlap between experimentally demonstrated SIRT interactors obtained from Biogrid (A). Deregulated SIRT interactors at the OT identified by proteomics across experimental groups (B).

**Additional file 12: Figure S6.** Bar graph representing the relative intensity of all bands observed by Western-blotting against Lys-acetylated proteins for each group (control, AD and PD) and olfactory region (OB, OT, EC and amygdala) in women and men after normalization to total stain protein. Data are presented as mean ± SEM. *P < 0.05 vs. control group; ***P < 0.001 vs. control group (a.u: arbitrary units).

**Additional file 13: Figure S7.** Altered OT biofunctions considering sex- and neuropathological dimensions. Braak (V–VI) and neocortical stages were considered in AD and PD, respectively.

## Data Availability

Mass-spectrometry data and search results files were deposited in the Proteome Xchange Consortium via the JPOST partner repository (https://repository.jpostdb.org) [101] with the identifier PXD038061 for ProteomeXchange and JPST001921 for jPOST (for reviewers: https://repository.jpostdb.org/preview/1400199357636bce4231af5 Access key: 8609). According to recent recommendations [102], sex annotation has been included in raw files to facilitate further analysis.
