# Supplementary figures and images for: Sex-divergent effects on the NAD+-dependent deacetylase sirtuin signaling across the olfactory–entorhinal–amygdaloid axis in Alzheimer’s and Parkinson’s diseases
Source: Biol Sex Differ. 2023 Feb 8;14:5. doi: 10.1186/s13293-023-00487-x (PMC9906849; doi:10.1186/s13293-023-00487-x)

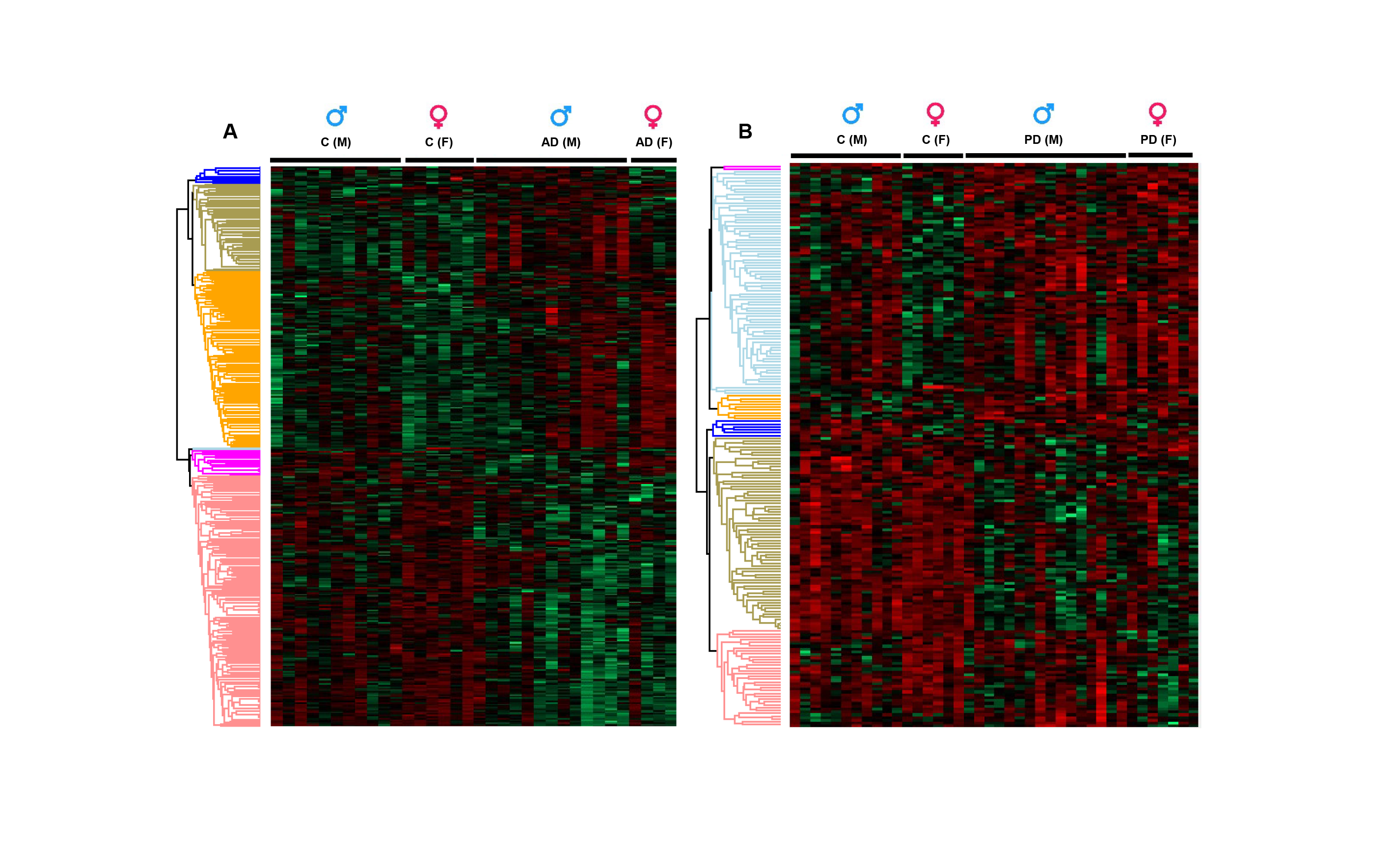

Supplement: Supplementary file 7 — Additional file 7: Figure S1. Sex-dependent clustering based on the olfactory tract (OT) protein expression profiles derived from AD (A) and PD (B) subjects. Heatmap representation showing both clustering and the intensity for the OT proteins in each biological condition (ANOVA p value). [file 13293_2023_487_MOESM7_ESM.tif]

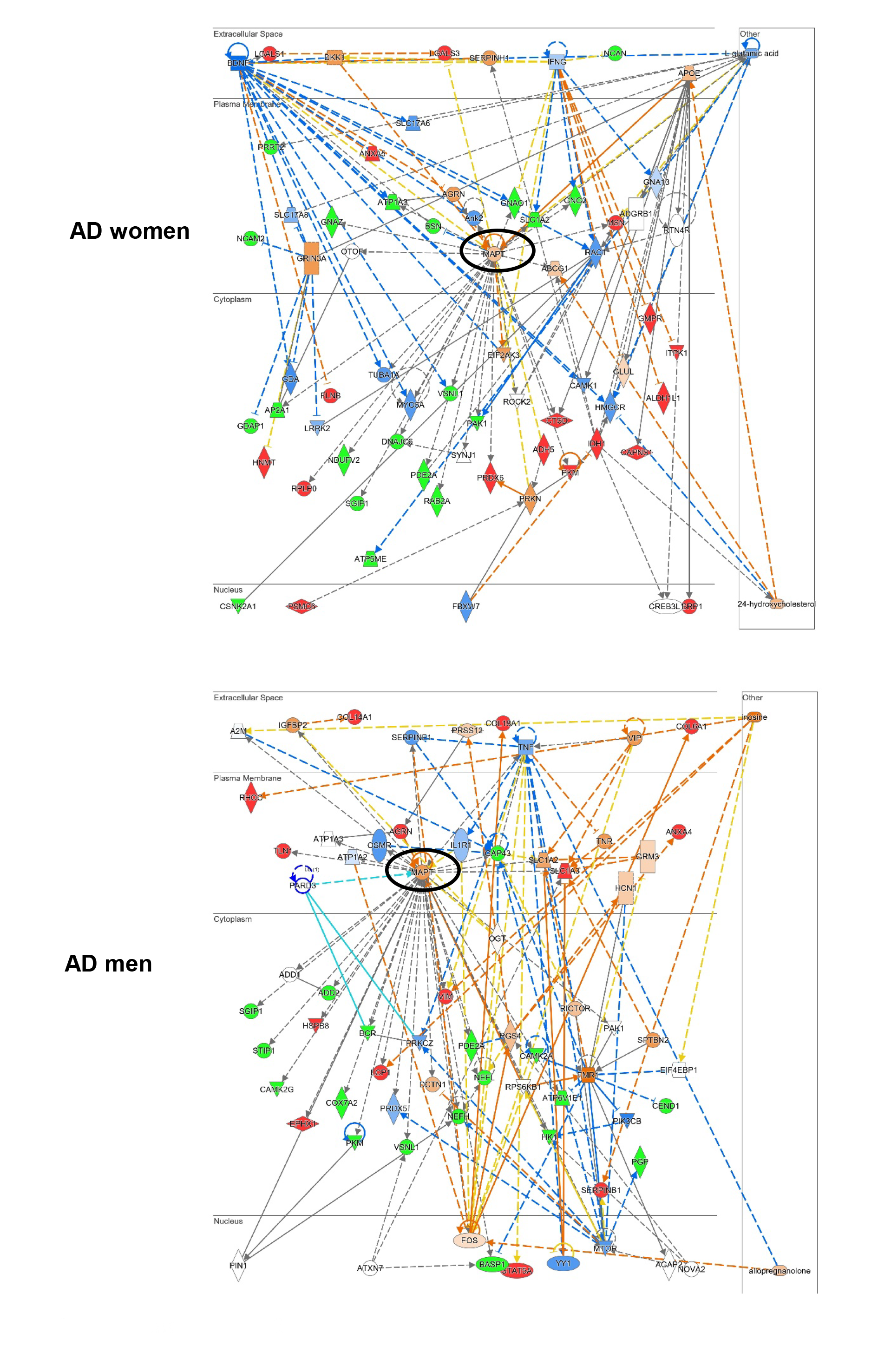

Supplement: Supplementary file 8 — Additional file 8: Figure S2. Deregulated protein interactome associated with MAPT (Tau) in AD generated by IPA software. Green and red indicate down and up-regulated proteins, respectively. Orange and blue indications are activation or inhibitory mechanisms proposed by the IPA algorithm. [file 13293_2023_487_MOESM8_ESM.tif]

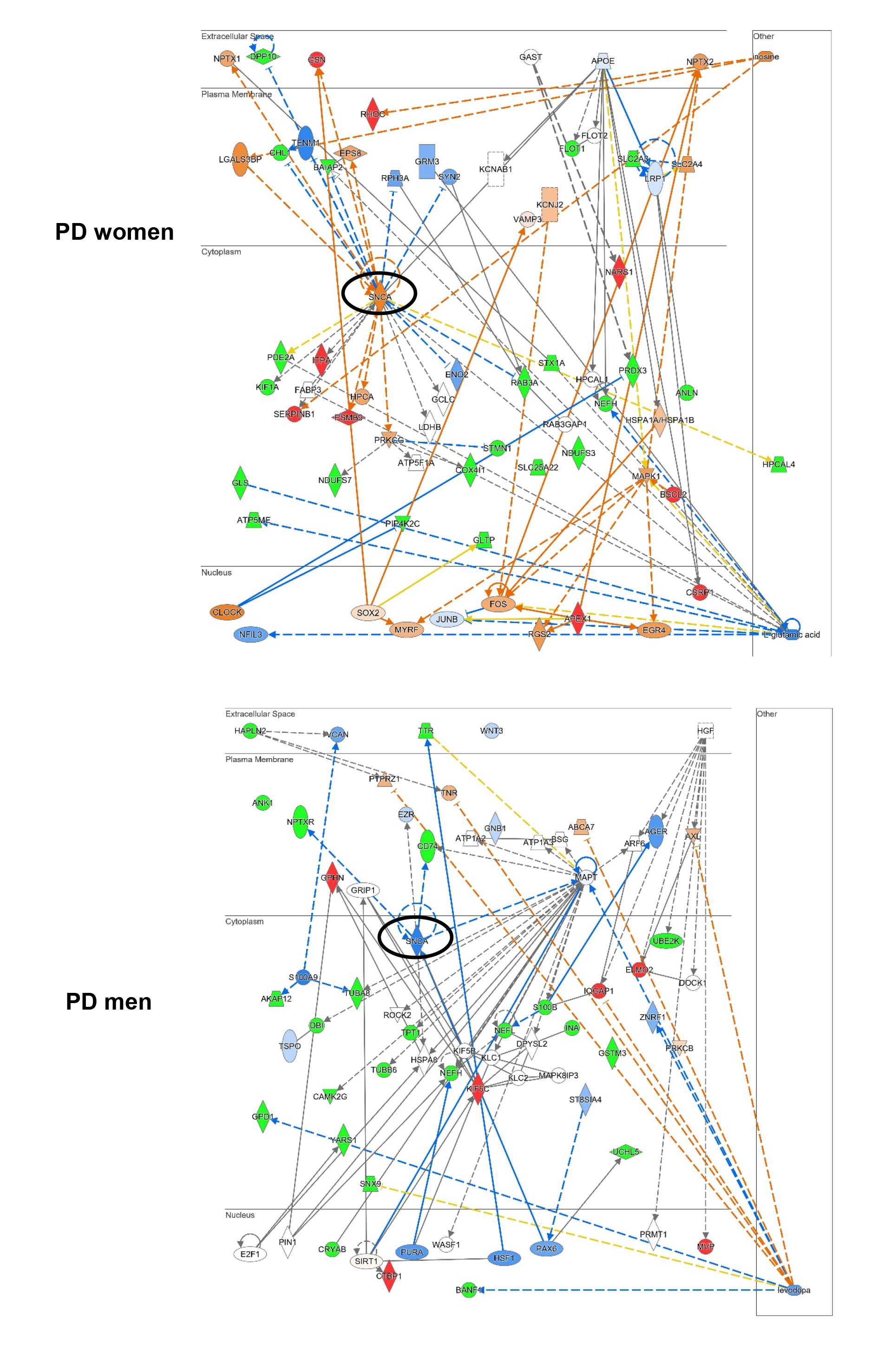

Supplement: Supplementary file 9 — Additional file 9: Figure S3. Deregulated protein interactome associated with SNCA (α-synuclein) in PD generated by IPA software. Green and red indicate down and up-regulated proteins, respectively. Orange and blue indications are activation or inhibitory mechanisms proposed by the IPA algorithm. [file 13293_2023_487_MOESM9_ESM.tif]

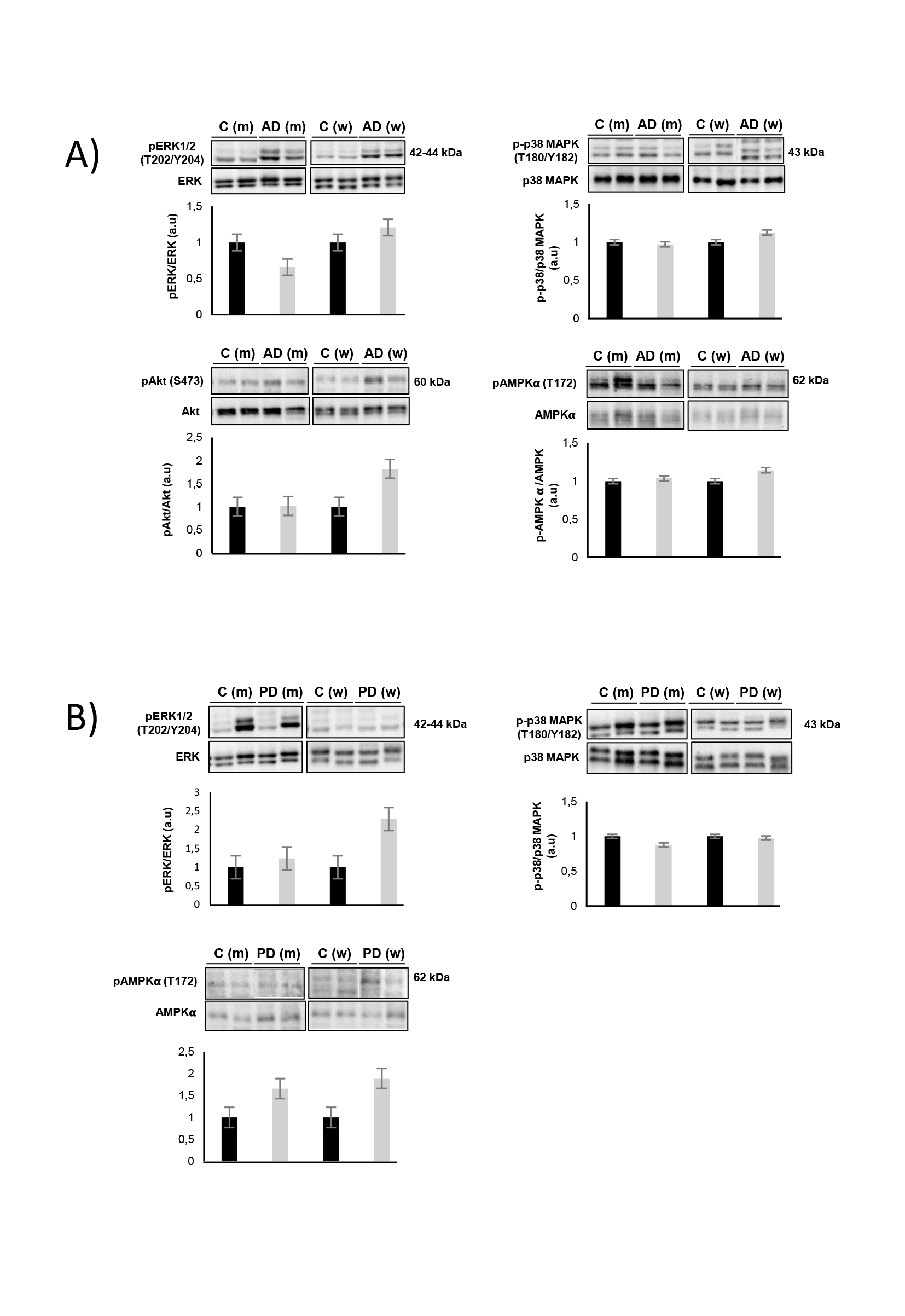

Supplement: Supplementary file 10 — Additional file 10: Figure S4. Western-blotting analysis of several survival kinases that are not modified in AD (A) or PD (B) at the level of the OT. Representative blots are shown. [file 13293_2023_487_MOESM10_ESM.tif]

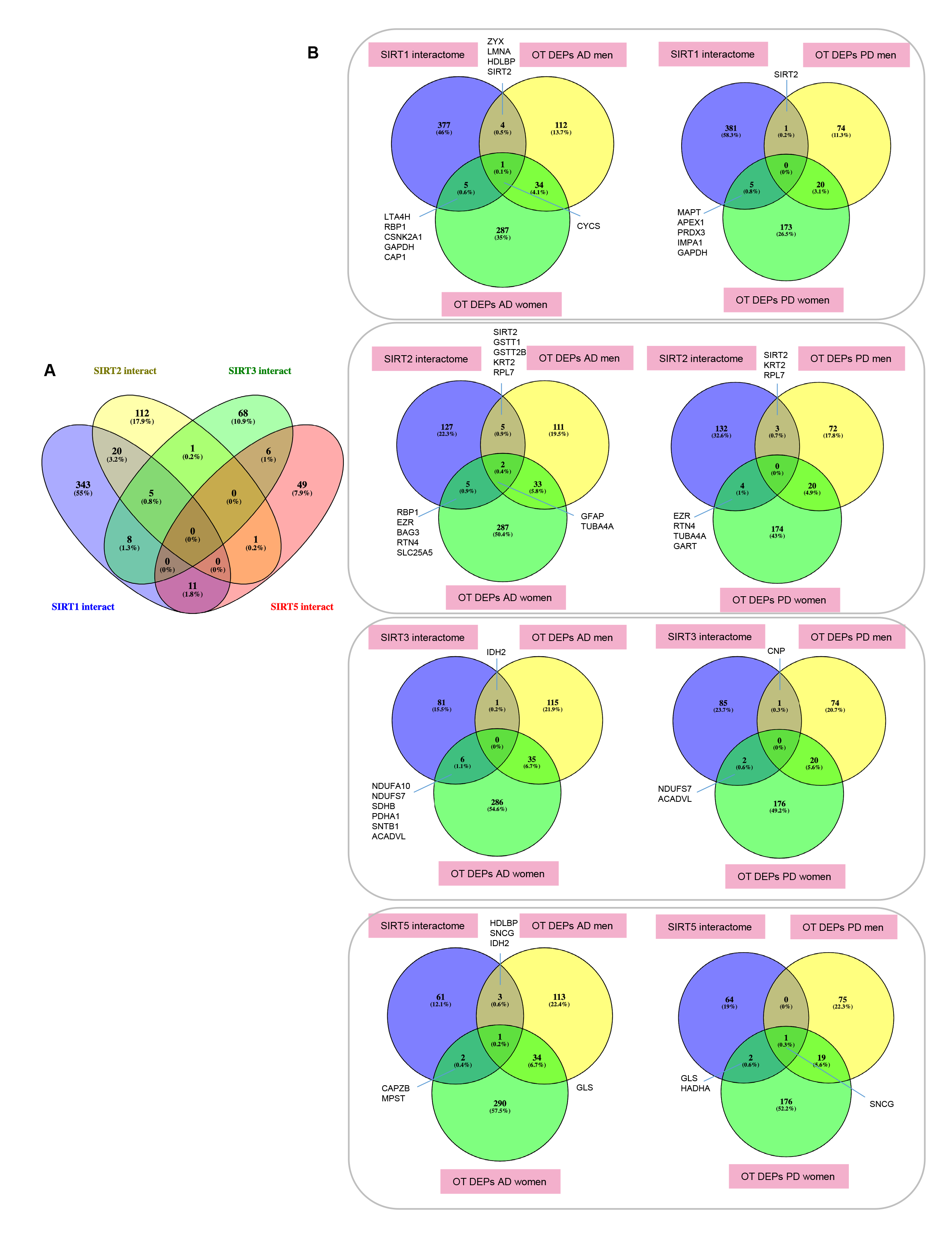

Supplement: Supplementary file 11 — Additional file 11: Figure S5. Exploration of human SIRT interactomes. Overlap between experimentally demonstrated SIRT interactors obtained from Biogrid (A). Deregulated SIRT interactors at the OT identified by proteomics across experimental groups (B). [file 13293_2023_487_MOESM11_ESM.tif]

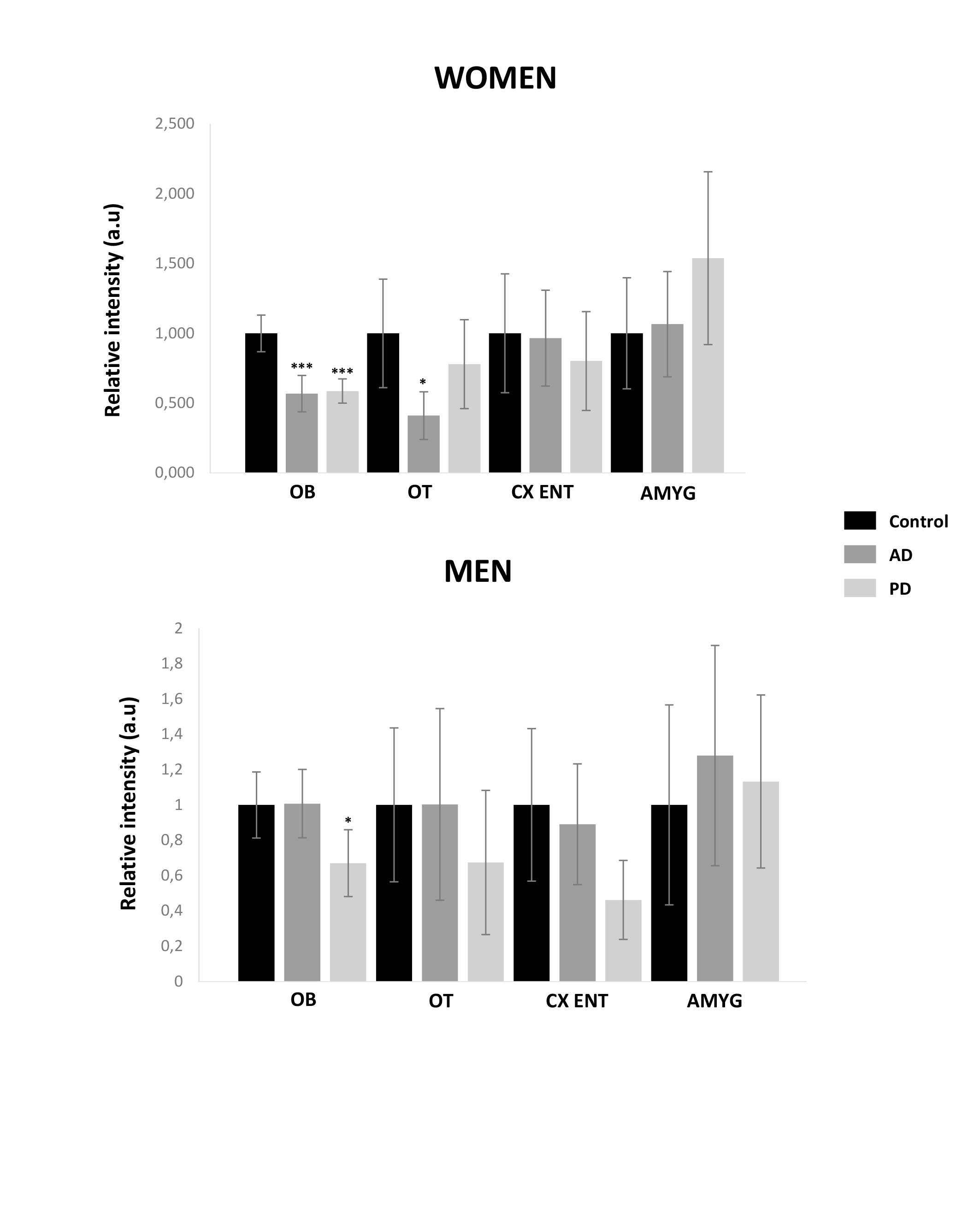

Supplement: Supplementary file 12 — Additional file 12: Figure S6. Bar graph representing the relative intensity of all bands observed by Western-blotting against Lys-acetylated proteins for each group (control, AD and PD) and olfactory region (OB, OT, EC and amygdala) in women and men after normalization to total stain protein. Data are presented as mean ± SEM. *P < 0.05 vs. control group; ***P < 0.001 vs. control group (a.u: arbitrary units). [file 13293_2023_487_MOESM12_ESM.tif]

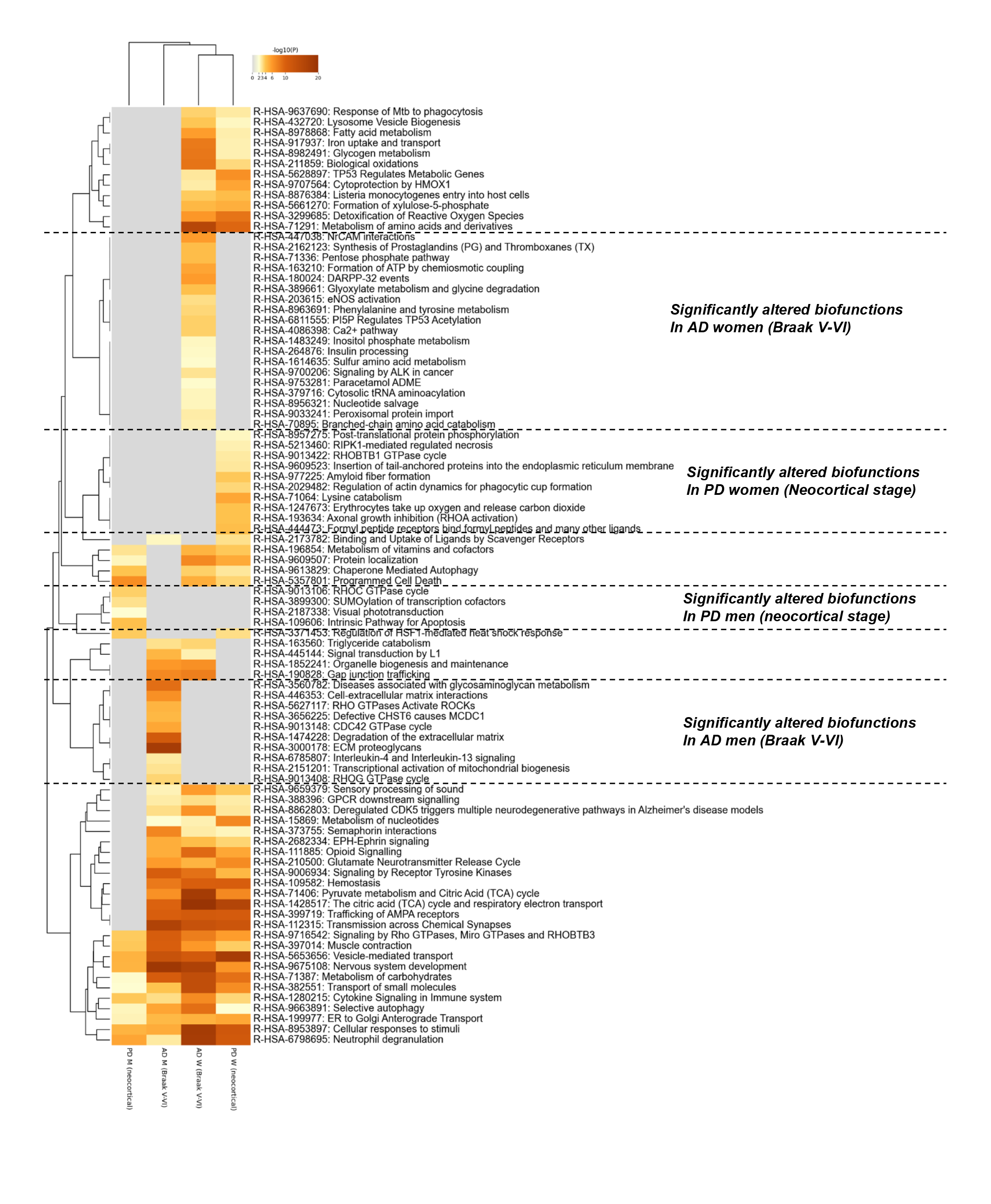

Supplement: Supplementary file 13 — Additional file 13: Figure S7. Altered OT biofunctions considering sex- and neuropathological dimensions. Braak (V–VI) and neocortical stages were considered in AD and PD, respectively. [file 13293_2023_487_MOESM13_ESM.tif]
